# Use of Arc Furnace Slag and Ceramic Sludge for the Production of Lightweight and Highly Porous Ceramic Materials

**DOI:** 10.3390/ma15031112

**Published:** 2022-01-31

**Authors:** Gamal A. Khater, Bassem S. Nabawy, Amany A. El-Kheshen, Manal Abdel-Baki Abdel Latif, Mohammad M. Farag

**Affiliations:** 1 Glass Research Department, National Research Centre, Cairo 12622, Egypt; aelkheshen1@yahoo.com (A.A.E.-K.); manalbaki@yahoo.com (M.A.-B.A.L.); mmfaragnrc@gmail.com (M.M.F.); 2 Geophysical Sciences Department, National Research Centre, Cairo 12622, Egypt; bsnabawy@yahoo.co.uk

**Keywords:** industrial wastes, arc furnace slag, ceramic sludge, β-wollastonite, parawollastonite, porous ceramics

## Abstract

The utility of recycling some intensive industries’ waste materials for producing cellular porous ceramic is the leading aim of this study. To achieve this purpose, ceramic samples were prepared utilizing both arc furnace slag (AFS) and ceramic sludge, without any addition of pure chemicals, at 1100 °C. A series of nine samples was prepared via increasing AFS percentage over sludge percentage by 10 wt.% intervals, reaching 10 wt.% sludge and 90 wt.% AFS contents in the ninth and last batch. The oxide constituents of waste materials were analyzed using XRF. All synthesized samples were investigated using XRD to detect the precipitated minerals. The developed phases were β-wollastonite, quartz, gehlenite, parawollastonite and fayalite. The formed crystalline phases were changed depending on the CaO/SiO_2_ ratio in the batch composition. Sample morphology was investigated via scanning electron microscope to identify the porosity of the prepared ceramics. Porosity, density and electrical properties were measured; it was found that all these properties were dependent on the composition of starting materials and formed phases. When increasing CaO and Al_2_O_3_ contents, porosity values increased, while increases in MgO and Fe_2_O_3_ caused a decrease in porosity and increases in dielectric constant and electric conductivity. Sintering of selected samples at different temperatures caused formation of two polymorphic structures of wollastonite, either β-wollastonite (unstable) or parawollastonite (stable). β-wollastonite transformed into parawollastonite at elevated temperatures. When increasing the sintering temperature to 1150 °C, a small amount of fayalite phase (Fe_2_SiO_4_) was formed. It was noticed that the dielectric measurements of the selected sintered samples at 1100 °C were lower than those recorded when sintering temperatures were 1050 °C or 1150 °C.

## 1. Introduction

The growth of industrial waste materials has become a significant public and ecological problem due to enlarged populations and rising industrialization. Solid waste handling is one of the most critical environmental issues in many developing countries. Solid waste is the byproduct of human activities such as the construction, utilization, and sharing of various resources in the world. Many studies have considered various aspects such as technology, innovation, recycling of solid waste management in growing and urbanized countries [1,2,3,4].

These wastes present many severe problems related to storage, shipping, and atmosphere or environmental pollution. The use of industrial waste in the fabrication of concrete, building materials, and cement is essential for hindering environmental pollution, lowering production expenses, and saving energy consumption. Thus, developing many technologies for recycling such waste is of great importance [5].

Among all industries, metallurgical industries are significantly costly to operate. Their byproducts are also the most abundant industrial wastes. The many kinds of waste products, such as junk, slags, and refractory wastes, make them a major waste-producing industry. Research by Mukhamedzhanova et al. [6] showed that the addition of accurate quantities of modifiers to metallurgical wastes could allow for the fabrication of many kinds of building materials.

Ferrous slag is a main waste byproduct obtained from iron and steel making. Various investigations have considered the use of iron slag as an economical raw material [7,8,9]. Many countries are interested in manufacturing construction materials via iron slag recycling. In Italy, 25–40 wt.% iron slag was added to clay to synthesize wall tiles. In South Africa, iron slag was used (up to 75 wt.%) in batches to obtain floor tiles with a water absorption ability of approximately 0.2 wt.% and bending strength of 43.8 MPa [10,11,12].

Arc furnace slag (AFS) is a byproduct of the steelmaking procedure, resulting from the use of electric arc furnaces, which account for more than 40% of the global steel production. AFS is created after melting and preliminary acid refining of liquid steel. It is a rocky and non-rugged material that can be crushed for use as aggregate in concrete batches [13,14,15].

A basic assumption is that AFS has an excellent prospect for use as a raw material in ceramic production on account of its aluminosilicate content and the matching of alkali and alkaline earth ingredients via their fluxing function in a prepared frit outline. These qualities can assist in the technical procedures of established processing. Ceramic wall and floor tiles are good candidates for producing controlled porosity bodies. Therefore, it is greatly successful to manufacture such ceramics through recycling of industrial byproducts [16].

Earlier researchers found that blast furnace slag was primarily anticipated to be an additive in Portland cement [17]. This was because of its pozzolanic and cementitious properties. By comparison, AFS does not have these features, and instead has higher iron content [18]. Pioro et al. [19] recommended that metallurgical slag be used to generate construction materials such as ceramic tiles. A complete combination of clays and AFS results in a balanced composition where the most wanted superior properties of tiles may be obtained by forming anorthite and wollastonite phases [20]. Wollastonite, also known as calcium silicate (CaSiO_3_), has been broadly studied due to its enormous applications in ceramics, dental inserts, structural design, and building, where it is used as a floor material as an alternative to granite and natural marble [21,22]. The most interesting character of this material is its ability to produce both curved and flat sheets [23].

Many efforts were made in earlier studies to build ceramic tiles employing 30–40 wt.% of AFS [18]. Teo et al. [16] established that the use of 40 wt.% AFS was capable of producing excellent mechanical properties in ceramic tiles [24].

The most widespread waste material produced from ceramic manufacturing is ceramic sludge. Several familiar artificial ceramics include wall tiles, floor tiles, sanitary ware, domestic ceramics, and traditional pottery. They are frequently created utilizing natural materials including clay and minerals. Ceramic wastes are classified into two groups according to the raw materials used [25]. The first group is wastes created by structural ceramic industries employing only red pastes for creating blocks, bricks, and roof tiles. The second category is fired ceramic wastes, which are formed in production of stoneware ceramic (floor and wall tiles and sanitary ware). Studies have revealed that during ceramic manufacturing, approximately 30% of the matter is wasted [26,27], and presently these wastes are not constructively reused, which causes much pollution to the environment. This indicates the need for research into pioneering methods of reusing ceramic wastes [28,29]. Ceramic sludge typically has high contents of SiO_2_ and Al_2_O_3_ oxides [30].

The recycling of ceramic sludge and electric arc furnace slag to produce ceramic tile material depends on the reaction between the SiO_2_ and Al_2_O_3_ of the ceramic sludge and the CaO of AFS, enhancing the properties of ceramic tile by developing calcium-aluminate-silicate crystals, such as anorthite and wollastonite. Therefore, it is a prominent way to cancel out the negative effect of iron oxide content in AFS on the densification process. This recycling process has many advantages in terms of cleaning the environment, storing energy, and using sustainable virgin materials [31].

The main goal of this study is to advance the use of two industrial wastes, arc furnace slag and ceramic sludge, for production of lightweight and porous ceramic materials that can be used for environmentally friendly construction. To fulfill this goal, ceramic samples containing parawollastonite, β-wollastonite and gehlenite minerals were prepared at different sintering temperatures. Microstructural, morphological, electrical, and some physical properties of those samples were studied.

## 2. Experimental Techniques

### 2.1. Batch Calculation and Samples Preparation

Nine ceramic samples were prepared from arc furnace slag (Al-Ezz-Dekheila Iron Steel Company, Alexandria, Egypt) and ceramic sludge (Ceramica Venezia, Cairo, Egypt). The arc furnace slag content in their composition varied from 10 wt.% to 90 wt.% at 10 wt.% intervals, and accordingly, the ceramic sludge content varied from 90 wt.% to 10 wt.%. The ceramic batches were prepared by calculating the appropriate proportions of arc furnace slag and ceramic sludge. The required silica and calcium oxide for all compositions were obtained from silica sand and limestone, respectively. The other minor elements present in the industrial wastes were considered during batch calculation. X-ray fluorescence (XRF) was used for chemical analysis of both waste materials (Table 1). The ceramic sample compositions were calculated based on ceramic sludge percentage with each successive increase in arc furnace slag percentage. These samples were designated AFS1, AFS2, AFS3, AFS4, AFS5, AFS6, AFS7, AFS8, and AFS9, as listed in Table 2.

**Figure 1:**
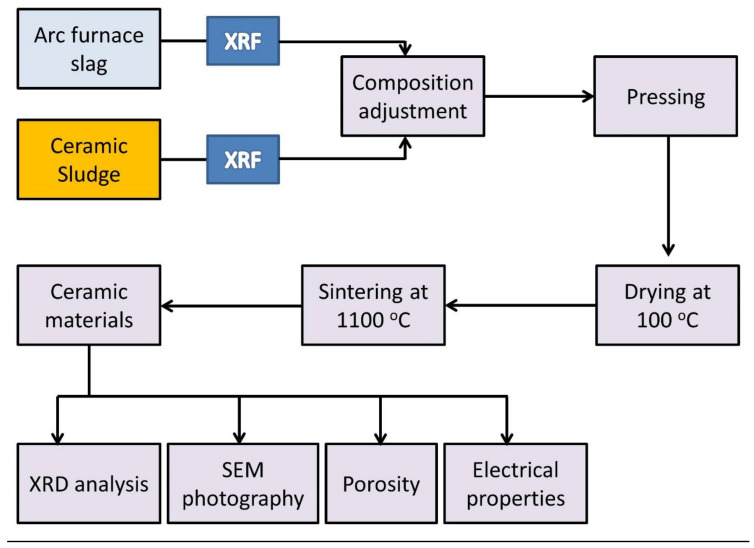
Schematic presentation of ceramic production from industrial wastes.

**Figure 2:**
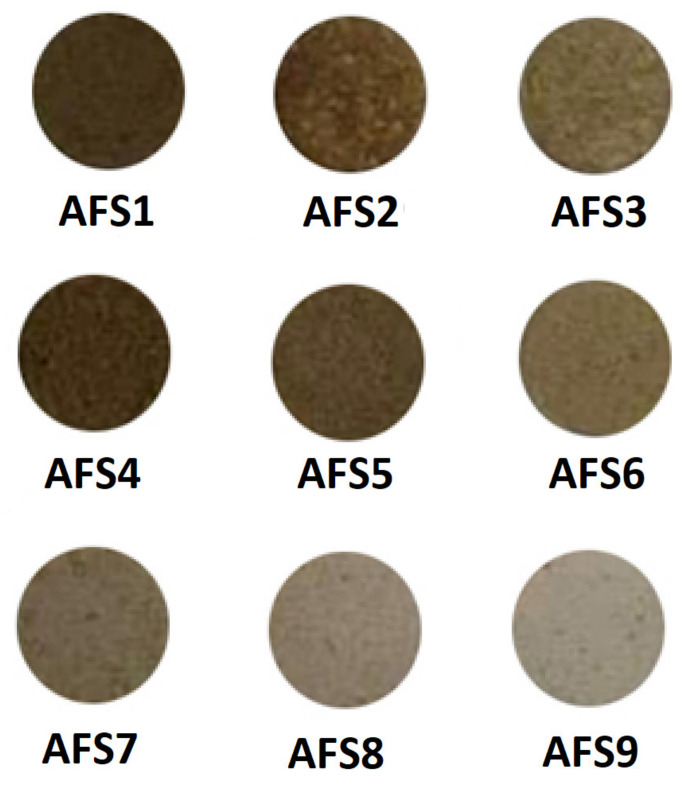
The surface appearance of ceramic samples prepared from industrial wastes after being treated at 1100 °C for 1 h.

Approximately 10 kg, as representative samples, of arc furnace slag, ceramic sludge, limestone, and silica sand were collected and crushed into −100 mesh powders. An adequate representative quantity for all stages of the laboratory investigation was obtained using the quartering technique. The prepared ceramic batches, after being accurately weighed to yield approximately 4 g for each sample, were closely dry-mixed in a ball mill for approximately 40 min until they became utterly homogeneous. Approximately 5% water was added to each sample as a binder, and then the powder samples were shaped into cylinders of 40 mm diameter and 4 mm thickness via uniaxial pressing at 20 MPa. The prepared ceramic batches were dried in an oven at a temperature of 100 °C for 24 h and then sintered at 1100 °C for one hour; this is shown in Figure 1. Figure 2 exhibits the visual appearance of the prepared samples.

Sample AFS5 was chosen, representing the average composition of all other samples, to study the effect of different sintering temperatures on the developed phases and microstructures.

### 2.2. X-ray Diffraction

The precipitated phases were identified with X-ray diffraction using a Bruker AXA diffractometer (D8—ADVANCE, Bruker, Germany) with Cu–Ka radiation, operating at 40 Kv, 40 mA, and a scanning rate of 10°/min.

### 2.3. Microstructure Photographs

The microstructure of the prepared ceramic samples was investigated utilizing a scanning electron microscope (SEM: JEOL, XL30, Philips, Amsterdam, Netherlands), which operated at an acceleration voltage of 20 kV. Each freshly broken sample was coated with a gold layer on the fracture surface (to reduce any charging effect) in order to observe the internal microstructure.

### 2.4. Porosity and Density Measurements

The dimensions, bulk volume (v_b_), and dry weight (w_d_) of the prepared samples were measured directly. For measuring weight and dimensions, a digital balance with 0.1 mg precision and a highly precise caliper sensitive to 0.01 mm were used, respectively. Then, the bulk density (ρ_b_) was estimated.

The grain density (ρ_g_) of the studied samples was measured using a helium pycnometer at the ambient conditions. Then, the helium porosity (∅_He_) was estimated considering the bulk and grain densities, as follows [32,33,34]:

∅_He_ (%) = 100 × (ρ_g_ − ρ_b_)/ρ_g_ (1)

Density measurements were taken five times for each sample, and the average value was calculated.

### 2.5. Dielectric and Electric Measurements

For the present samples, the capacitance effect (*Cp*), electric loss tangent (*D*), and electric resistance (*Rp*) were measured in a parallel series using a computerized LCR Hitester impedance bridge (IM3536) in the range of 50 Hz–8 MHz at 350 AC frequency points. Then, the dielectric constant (*ε*’), dielectric loss (*ε*”), imaginary part (*M*”) of the complex electric modulus, and electric conductivity (*σ*) were estimated as follows:

(2) $$\sigma =\frac{L}{A\times R_{P}}$$

(3) $$\varepsilon \prime =\frac{C_{P}\times L}{A\times \varepsilon_{0}}$$

*ε*” = *ε*’ × *D* (4)

(5) $$M\prime \prime =\frac{\varepsilon \prime \prime }{\varepsilon \prime^{2}+\varepsilon {\prime \prime }^{2}}$$

where *ε*’ is the dielectric constant, *Cp* is the electric capacitance in parallel, *L* is the sample thickness, *A* is the contact area of the measuring non-polarized electrode, *ε*” is the dielectric loss, *ε_ο_* is the dielectric constant of the air (8.85 × 10^−12^ F/m), *D* is the measured electric loss tangent (tan *δ*), R_P_ is the measured electric resistance in parallel, *σ* is the electric conductivity, and *M*” is the imaginary electric modulus.

More details on the applied techniques are discussed by many authors [35,36,37].

For determining the implications of different sintering temperatures on the electric and dielectric properties of the studied samples, three samples of the same composition (50% arc furnace ceramic slag + 50% sludge; AFS5), were sintered at 1050 °C, 1100 °C, and 1150 °C. Then, their density, porosity, and electric and dielectric parameters were measured.

## 3. Results and Discussions

### 3.1. X-ray Diffraction Patterns

**Figure 3:**
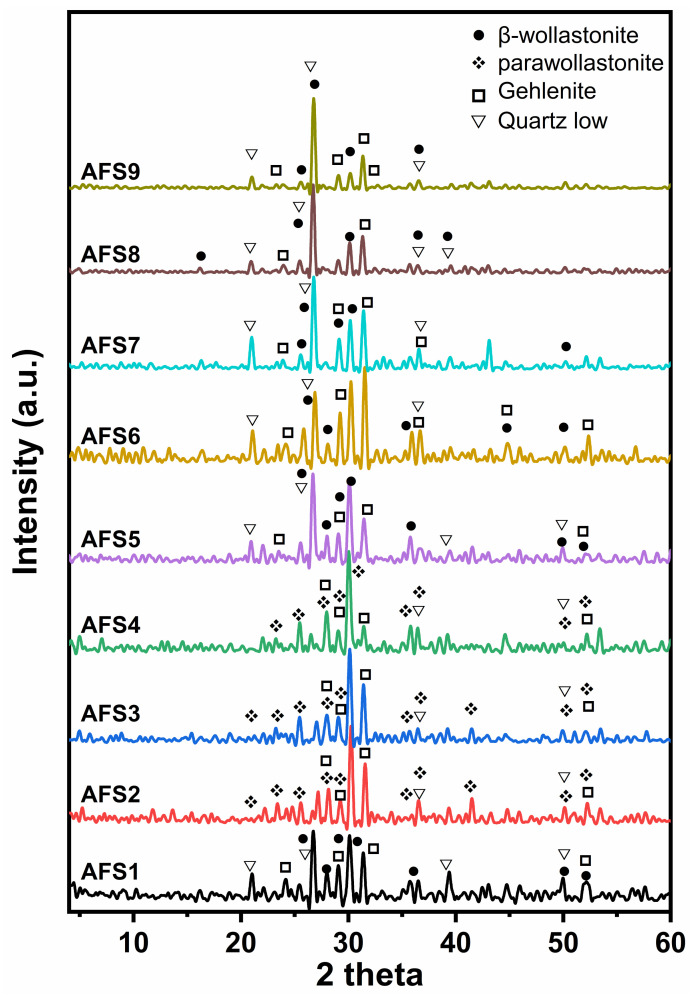
The major phases resulting from industrial wastes, determined via XRD after treatment at 1100 °C for one hour.

X-ray diffraction graphs (Figure 3) of investigated ceramic samples AFS1–AFS9, after sintering at 1100 °C for one hour, showed all precipitated phases of the samples. The major crystalline phases formed were β-wollastonite (CaSiO_3_) (JCPDS No.29-372), parawollastonite (CaSiO_3_) (JCPDS No.27-88), gehlenite (Ca_2_Al_2_SiO_7_) (JSPDS No.35-755), and low quartz (SiO_2_) (JCPDS No.5-0490). Sample AFS1 contained β-wollastonite (CaSiO_3_) as the dominant phase with lines at 3.33, 2.96, 3.16, and 3.06 Å. Low quartz (SiO_2_), which has distinctive lines 4.23, 3.33, 2.29, and 1.82 Å, represented the second phase. Gehlenite (CaAl_2_SiO_7_) was the third phase with lines at 3.72, 2.85, 2.43, 2.04, and 1.75 Å. Samples AFS2–AFS4 showed a significant decrease in quartz phase content. In addition, it is noticed that there was a transformation of β-wollastonite into parawollastonite, as seen through reduction in line heights at 3.33 and 2.51 Å corresponding to β-wollastonite and increases in intensity for the main lines of parawollastonite, which are 2.96, 3.16, and 3.83 Å; thus, parawollastonite was the main phase, and gehlenite was considered the second phase with small amounts of low quartz. In sample AFS5, β-wollastonite and low quartz phases reappeared with lines at 3.33, 2.96, 3.16, and 3.06 Å for β-wollastonite and lines at 4.23 3.33, 2.29, and 1.82 Å for low quartz. Sample AFS6 showed the development of gehlenite phase, which became the main phase with increasing intensities of lines at 2.85, 3.72, 2.43, 2.04, and 1.75 Å. AFS6’s pattern indicated the formation of β-wollastonite and quartz, while samples AFS7, AFS8, and AFS9 showed development of both low quartz and β-wollastonite phases and a noticeable decrease in the intensity of gehlenite lines at 3.72, 2.85, 2.43, 2.04, and 1.75 Å.

Through a deep glance at Table 2, it can be found that sample AFS1 contained a CaO/SiO_2_ ratio of approximately 0.68, which confirms the X-ray diffraction results. That accounted for β-wollastonite as a primary phase with low quartz and gehlenite as secondary phases. For samples AFS2–AFS4, the CaO/SiO_2_ ratio decreased from 0.63 to 0.50, corresponding with the transformation of β-wollastonite into parawollastonite. A decrease in the percentage of low quartz phase and an increase in the percentage of gehlenite were also noticed in samples AFS2, AFS3, and AFS4. The CaO/SiO_2_ ratio decreased to 0.41 in sample AFS5, which helped form β-wollastonite and gehlenite as the major phases and low quartz as secondary phase. For sample AFS6, the CaO/SiO_2_ ratio increased to 0.51; this led to the formation of gehlenite as the main phase with β-wollastonite and low quartz as secondary phases. Samples AFS7–AFS9 had nearly the same CaO/SiO_2_ ratio, approximately 0.52–0.53, which helped form β-wollastonite as the primary phase with low quartz as the second phase and gehlenite as the third phase. All the above results indicate that β-wollastonite (unstable phase) was formed more often than low quartz, with a small amount of gehlenite. The β-wollastonite phase turned into parawollastonite (stable phase) when the CaO/SiO_2_ ratio decreased. The parawollastonite phase was formed with greater amounts of gehlenite and smaller amounts of low quartz.

The obtained results indicate that a low CaO/SiO_2_ ratio enhances the formation of β-wollastonite and low quartz phases but hinders the development of gehlenite phases (samples AFS7–AFS9). At the same time, increasing the CaO/SiO_2_ ratio promotes the formation of parawollastonite and gehlenite phases but hinders the development of the low quartz phase (samples AFS2–AFS4).

Fan et al. [38] explained that by increasing the CaO/SiO_2_ ratio, the basicity increases, and consequently, the crystallization of the CaO-Al_2_O_3_-MgO-SiO_2_ system is improved [39,40]. Tabit et al. [41] found that parawollastonite and gehlenite were formed in samples containing higher calcium oxide percentages.

#### Effect of Sintering Temperatures on Crystalline Phases

**Figure 4:**
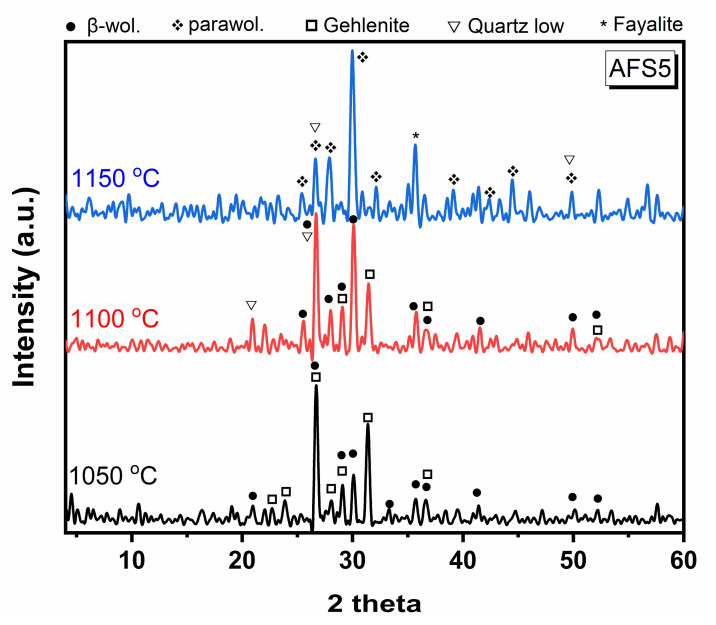
X-ray diffraction patterns of AFS5 samples after sintering at different temperatures for one hour.

AFS5 was chosen to study the effect of sintering temperatures on precipitated phases and resulting physical properties, because it represents the mean composition of all the examined samples. The studied sintering temperatures were 1050, 1100, and 1150 °C, all for one hour. Figure 4 shows that, after sintering at 1050 °C, β-wollastonite (CaSiO_3_) became a primary phase with its characteristic lines at 3.33, 2.967, and 2.51 Å. Low quartz (SiO_2_) was the second phase, with its distinctive lines at 4.24, 3.33, and 2.46 Å, and gehlenite (Ca_2_Al_2_SiO_7_) was the third phase, with its characteristic lines at 3.74, 3.17, and 2.84 Å. When the temperature was raised to 1100 °C, the gehlenite lines decreased, evident in the mainline 2.84 Å. In addition, β-wollastonite began to convert into parawollastonite. This transformation is demonstrated by the increasing intensity of the line at 2.96 Å and the decreasing intensity of the β-wollastonite line at 3.33 Å. Upon raising the temperature to 1150 °C, β-wollastonite transformed into parawollastonite, which is evident from the decreased intensity of its line at 3.33 Å and increased intensity of the parawollastonite line at approximately 2.97 Å. Gehlenite wholly disappeared, as its distinctive line at 2.84 Å vanished. This increase in sintering temperatures promoted the formation of the fayalite (Fe_2_SiO_4_) phase. These results agree with Edrees et al. [42], who showed that β-wollastonite is an unstable phase at lower temperatures and turns into parawollastonite (stable phase) at higher temperatures. Ismail et al. [43] found that wollastonite has the property of a polymorphic structure, whether as β-wollastonite (unstable) or parawollastonite (stable). β-wollastonite turns into an independent parawollastonite at high temperatures. Qin et al. [44] also showed that gehlenite was formed with wollastonite and disappeared at high temperatures (1125 °C). The effect of the CaO/SiO_2_ ratio on the phase transformation of anorthite, based on steel slag and fly ash, was studied by Tabit et al. [41]. They deduced that the gehlenite phase was precipitated at 1100 °C.

### 3.2. Morphology Studies

**Figure 5:**
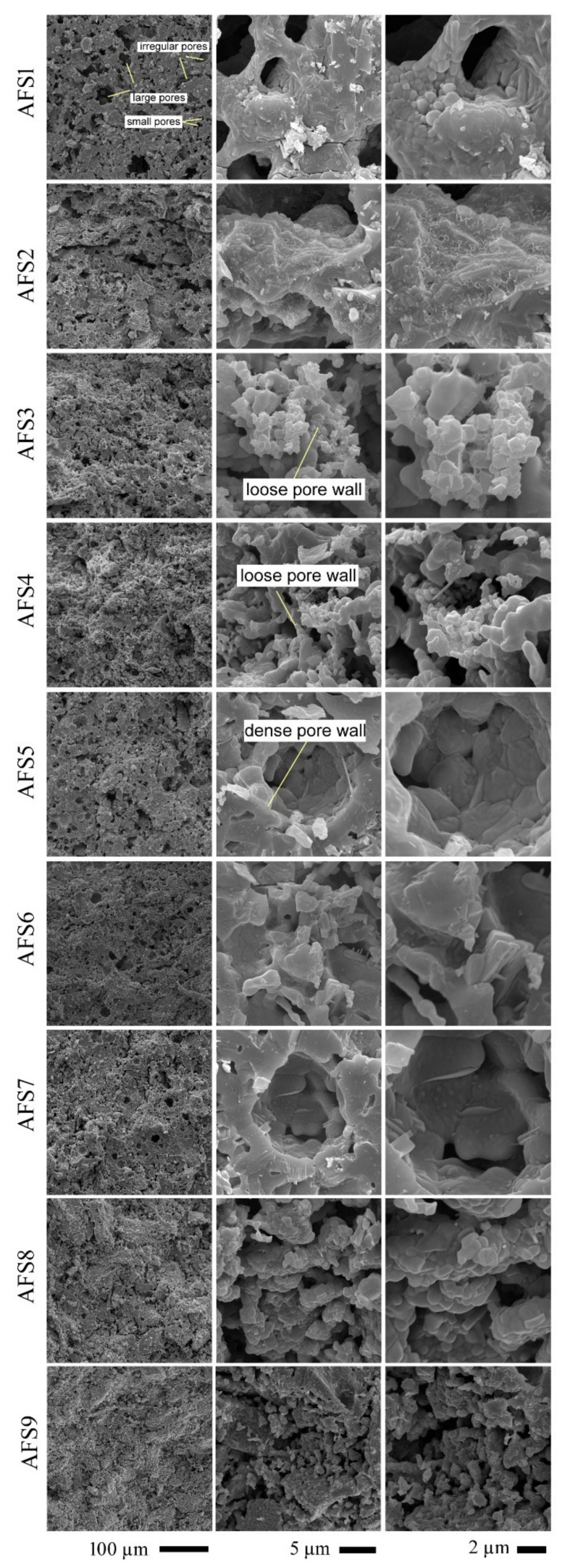
SEM images at different magnifications of microstructures of the investigated samples after treatment at 1100 °C for 1 h.

Figure 5 presents SEM photographs at different magnifications of the fractured samples AFS1–AFS8 after heat treatment at 1100 °C. It can be observed from the figure that all ceramic samples possessed porous structures, and there were wide variations in the pore size distribution (the pore diameter ranged from 4 to 80 μm) and shape. Circular and irregular pore outlines were noticed in all ceramic samples. Moreover, closed and interconnected pores are apparent in the photos. Porosity decreased and density increased with increases in arc furnace slag (AFS) weight, as is confirmed from the porosity percentage results (decreasing from 41% for AFS1 to 28% for AFS8), and the increasing density of wall pores (Figure 5). This can be attributed to the decrease in CaO and increase in MgO. As MgO molecules are smaller than CaO molecules, this caused a decrease in liquidus temperature, as reported in a previous study [45]. In addition, decreasing Al_2_O_3_ content caused a decrease in the liquidus temperature of the ceramic particles. When Al_2_O_3_ content increases, the amount of nonbridging oxygens (NBOs) decreases and consequently, Al–O–Si bonds form and raise viscosity values [46]. This decrease in liquidus temperature leads to more condensed ceramic samples with lower porosity. Moreover, columnar and tabular crystals, which are the characteristic structures of wollastonite crystals, were noticed.

#### Effect of Sintering Temperatures on Ceramic Morphology

**Figure 6:**
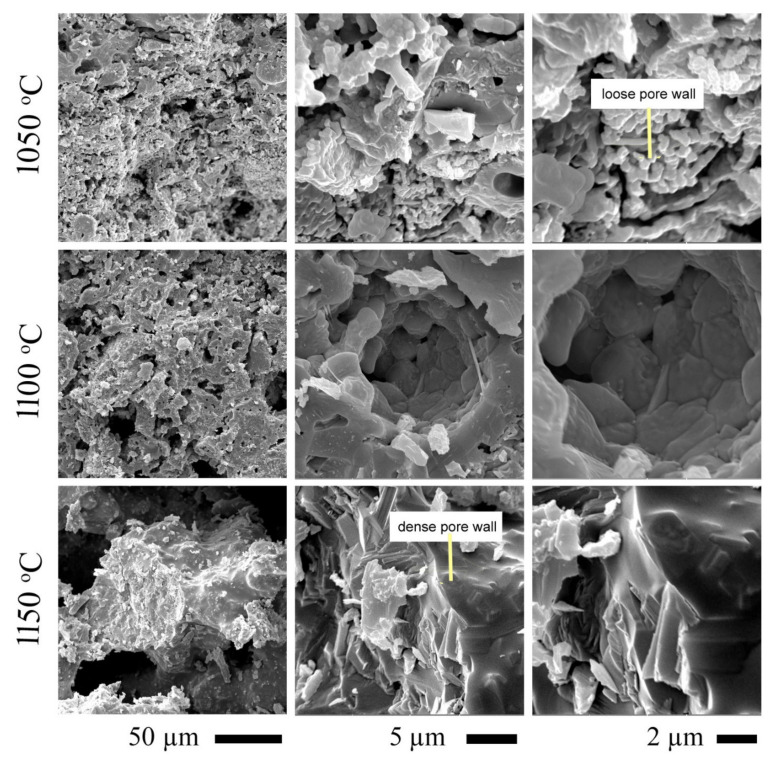
SEM images at different magnifications of microstructures of AFS5 ceramic sample after treatment at 1050 °C, 1100 °C, and 1150 °C.

The applied sintering temperatures for ceramic materials affect properties such as porosity, density, and microstructure. Accordingly, different sintering temperatures were applied on AFS5 to determine the optimum conditions for producing such ceramic. The sample was heat-treated at 1050 °C, 1100 °C, and 1150 °C at a constant time (Figure 6). As shown from the figure, an increase in sintering temperature from 1050 °C to 1100 °C led to a denser and more compact structure. This could be due to the formation of well-developed, dense, and long wollastonite crystals. Upon raising the sintering temperature to 1150 °C, a slight change in microstructure was noticed. This increase in temperature caused the appearance of new rod-shaped crystals with a minor increase in the porous structure and a loss of tiny pores. This might be because of the disappearance of the gehlenite phase and the formation of the fayalite phase, as well as due to the transformation of the β-wollastonite (triclinic) structure into the parawollastonite (monoclinic) structure.

### 3.3. Porosity and Density Studies

**Figure 7:**
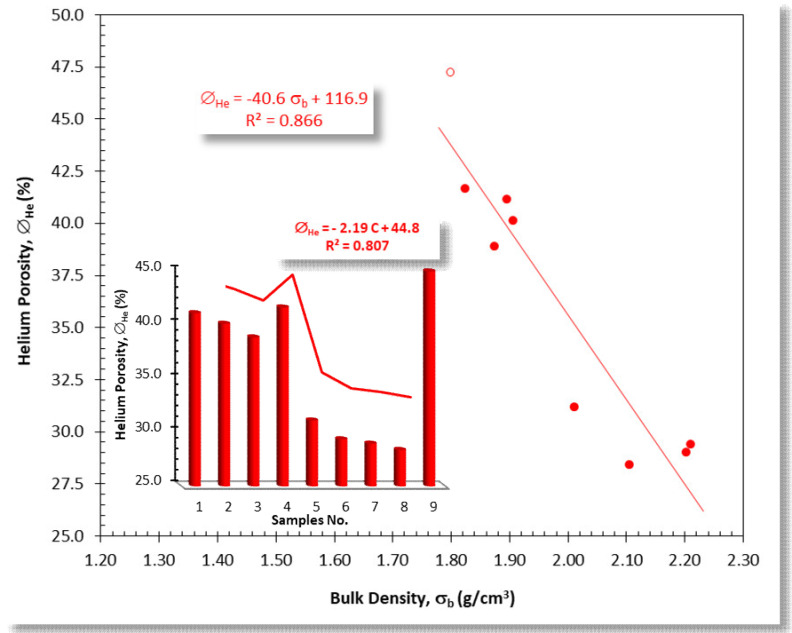
Plot showing the inverse proportional relationship between the bulk density (ρ_b_) and the helium porosity (∅_He_) of the AFS samples, and the frequency bars and broken line distribution. Open circle represents sample AFS9, which was removed from processing due to its abnormal porosity value.

The prepared samples are considered highly porous materials with very high helium porosity values (∅_He_) and shallow bulk density values (ρ_b_) (Table 3 and Figure 7). The lowest density values were primarily assigned to the first four samples AFS1–AFS4 and the ninth sample AFS9, which consisted primarily of monoclinic wollastonite, CaSiO_3_, ρ_g_ = 2.86–3.09 g/cm^3^, [47] and gehlenite, Ca_2_Al_2_SiO_7_, ρ_g_ = 3.04 g/cm^3^ [48], with some quartz content, SiO_2_, ρ_g_ = 2.62–2.65 g/cm^3^ [49] (Table 3).

The inverse proportional relationship between bulk density and porosity (Figure 7) is due to the high dependence of pore volume and grain density on the principal components of the material. In general, the porosity values decreased from AFS1 to AFS8 (with an exception for sample AFS4). Sample AFS9 showed an increase in porosity value. Therefore, porosity decreased as arc furnace slag content increased from 10% to 80%, in samples AFS1 and AFS8, respectively (Table 2, Figure 7). ∅_He_ can be estimated as a function of the bulk density and the arc furnace slag content, based on two mathematical models, as shown in Figure 7.

#### Effect of Sintering Temperatures on Porosity and Density Values

The implication of sintering temperatures on porosity was checked for sample AFS5, characterized by a median ratio of arc furnace slag to ceramic sludge (50% for each). Porosity decreased with increasing sintering temperature, which was due to the increasing crystallization grade and crystal size with increasing temperatures (Table 4).

Table 4 exhibits the porosity and density results of sample AFS5 after sintering at different temperatures, along with the precipitated phases at each temperature. This table shows that porosity decreased upon increasing the sintering temperature from 1050 °C to 1100 °C. This was because of the increase in compactness of the well-sintered sample. However, when sintering temperature was increased to 1150 °C, a slight increase in porosity and decrease in density was found, which can be explained by the transformation of β-wollastonite into parawollastonite as well as due to the complete vanishing of gehlenite. Although the fayalite phase with its high density value, 4.39 [50], was precipitated, the density of AFS5 sample decreased after sintering at 1150 °C. This may be due to its low content in the sample.

### 3.4. Electric and Dielectric Properties

**Figure 8:**
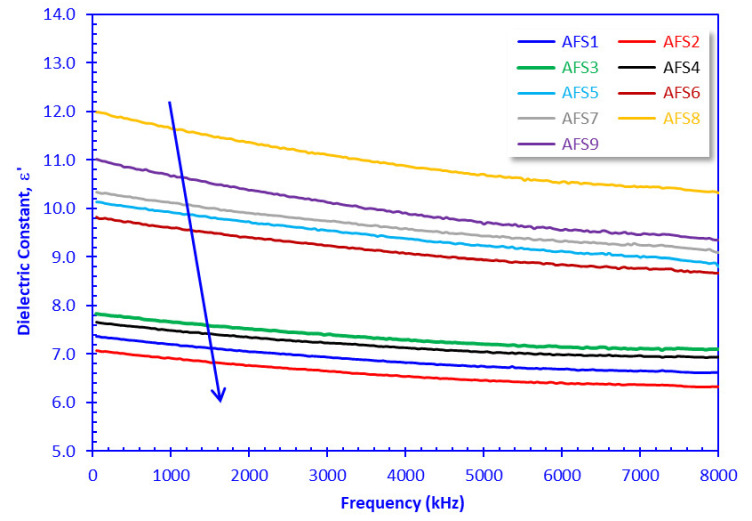
Plotting the dielectric constant of the samples versus the applied frequency.

**Figure 9:**
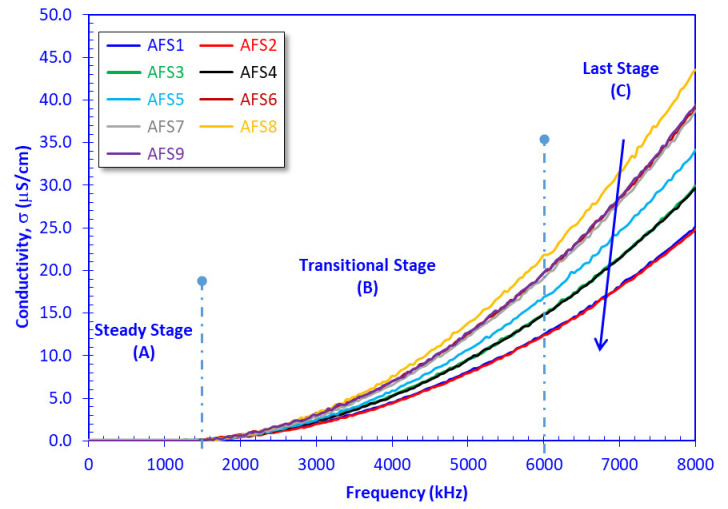
Plotting the electric conductivity of the samples versus the applied frequency.

The dielectric constant values (*ε*’) of the AFS samples fluctuated between 6.324 for sample AFS2 at 8 MHz and 11.988 for sample AFS8 at 50 Hz (Figure 8), while the electric conductivity values (*σ*) varied from 0.0105 µS/cm for sample AFS1 at 50 Hz to 43.57 µS/cm for sample AFS8 at 8 MHz (Figure 9). Based on the *ε*’ values, the AFS samples can be grouped in descending order into two groups: group 1 (AFS8, AFS9, AFS7, AFS5, AFS6 samples) and group 2 (AFS3, AFS4, AFS1, AFS2 samples), as can be seen in Figure 8. The dielectric constant decreased with increasing applied AC frequency, while electric conductivity increased dramatically with increasing AC frequency, a common behavior recorded by many authors [35,36,51]. Following Nabawy and Rochette [38], the increase in conductivity with increasing frequency passes through three stages: (A) a steady stage with negligible increase in *σ*, (B) a transitional stage, and (C) a last stage with a dramatic increase in *σ* (Figure 9).

**Figure 10:**
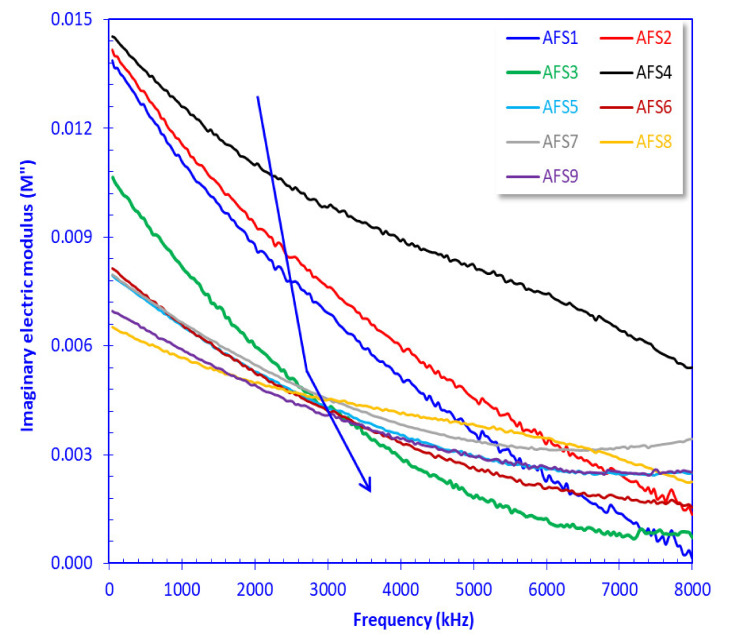
Plotting the imaginary part of the complex electric modulus of the samples versus the applied frequency.

The imaginary electric modulus for samples AFS4, AFS2, and AFS1 was relatively high at 50 Hz (0.1388–0.0145), whereas it was lower for the other samples with different slopes, causing an inflection point in the frequency range of 2.5–3.0 MHz (Figure 10). These different dipolar relaxations were attributed to different relaxations for *ε*’ and *ε*” at higher frequencies and to the crystal surfaces, which become more active at higher frequencies [52,53,54,55].

Based on the *σ* classification of Khater et al. [35,36], differentiation of *σ* values is difficult at low frequency values (*σ* = 0.01–0.181 µS/cm, *f* < 1500 kHz, Figure 9) and refers to poor semiconductors (0.01–1.0 µS/cm, Khater et al. [35,36]), whereas *σ* values are higher at high frequency values (*σ* = 0.181–43.57 µS/cm, *f* ≥ 1500 kHz) referring to poor (0.01–1.0 µS/cm) to fair semiconductors (1.0–1000 µS/cm, Khater et al. [35,36]). The measured electric and dielectric parameters are presented graphically as a function of the AC frequency in Figure 8, Figure 9 and Figure 10.

#### Effect of Sintering Temperatures on Electric Properties

**Figure 11:**
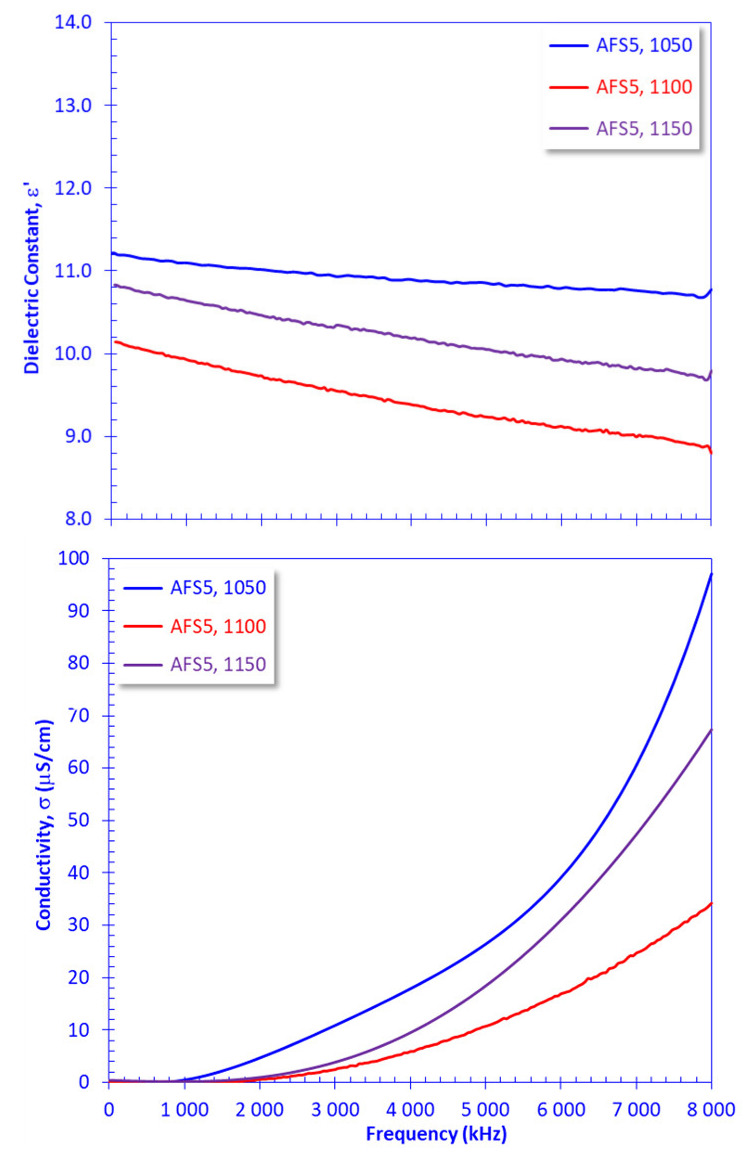
Effect of sintering temperature (1050–1150 °C) of AFS5 on the measured dielectric constant and electric conductivity plotted versus the applied frequency.

Increases in the crystallization grade were the primary variable affecting the electric and dielectric properties of sample AFS5, as shown in Figure 11. It is indicated that the crystal size was smaller for AFS5 sintered at 1050 °C. At 1100 °C, the crystal size was well-developed with reduced pore volume, which caused a decrease in the electric and dielectric parameters. Sintering at this temperature enhanced dielectric properties, such that it could be considered a more appropriate semiconductor material than the samples sintered at 1050 °C and 1150 °C (Figure 11).

### 3.5. Implication of Waste Composition on Pore Volume

**Figure 12:**
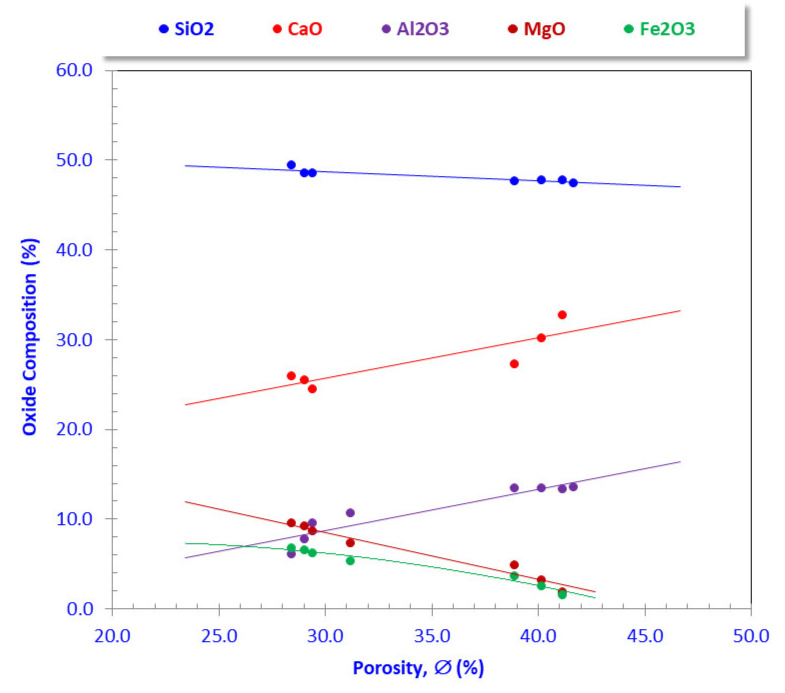
Plotting the main oxide composition versus the incremental percentages of porosity.

The incremental increase in CaO and Al_2_O_3_ fractions was accompanied by increasing porosity values, while MgO and Fe_2_O_3_ contents were inversely related to the porosity values. The impact of increasing SiO_2_ content acted somewhat inversely upon the porosity values (Table 3, Figure 12). Consequently, the decreasing sludge content from AFS1 to AFS9 was responsible for the decreasing pore volume of the studied samples (Table 2 and Table 3).

### 3.6. Implication of Waste Composition on Electric Properties

The waste composition of prepared ceramic samples impacted their electric activities. Electric conductivity and capacitance were primarily affected by the arc furnace slag content in an incremental increase from the AFS1 to the AFS9 samples (10–90%, respectively). Based on their dielectric and electric parameters, the AFS samples could be clustered into two groups, AFS1–AFS4 and AFS5–AFS9. These groups may be differentiated by the higher porosity values for the first group (41.12–41.66%) compared to the second group (31.16–47.26%, Table 3). This could be attributed to higher contents of CaO (32.73–23.79%) and Al_2_O_3_ (13.42–13.61%) in the first group (AFS1–AFS4) than the second one (Table 2).

Likewise, based on the dielectric constant values, the arc furnace slag–sludge mixtures could be clustered into two sample groups; the first one (AFS5–AFS9) was characterized by higher *ε*’ and *σ* and lower *M*” than the second (AFS1–AFS4) (Figure 8, Figure 9 and Figure 10). This could be attributed to the relatively high contents of Fe_2_O_3_ and MgO in samples AFS5–AFS9 and the relatively low contents of CaO and Al_2_O_3_ in these samples (Table 2).

### 3.7. Implications of Crystal Size and Pore Volume on Electric and Dielectric Properties

The crystal size had a direct effect on the electrical activity of the charged materials. Larger crystal sizes result in reduced surface area and, therefore, less polarized charged surfaces. For samples AFS5–AFS9, the crystals seemed to be well developed, as shown in Figure 5, while for samples AFS1–AFS4, the crystals had a lower surface area.

**Figure 13:**
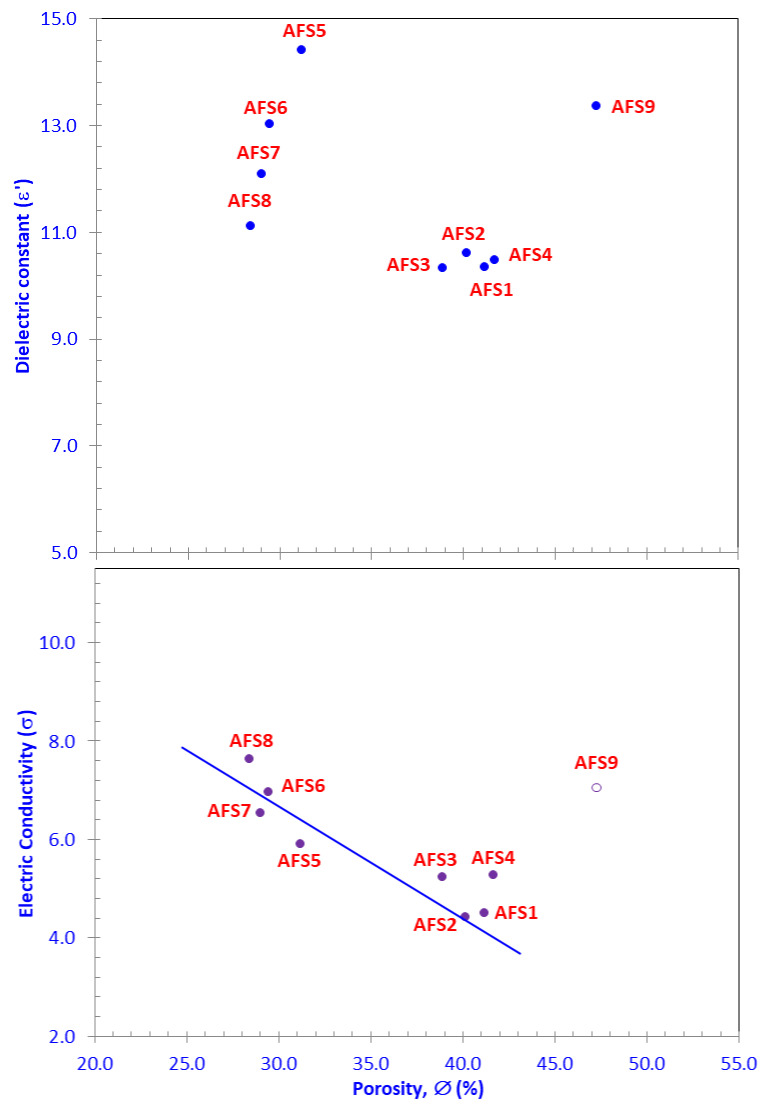
Dielectric constant (*ε*’) and electric conductivity (*σ*) versus helium porosity (∅_He_). Sample AFS9 (open circle) was removed from statistical processing.

By contrast, porosity (∅ in%) is a complementary component of the crystal’s volume (equal to 100–∅ in%), i.e., it has indirect effects on the studied samples [34,52,53]. Porosity is considered an additional parameter that controls the electrical polarization and conductivity of samples. High porosity means lower grain volume and less polarized surfaces, and vice versa. Samples AFS5-AFS8 had porosity values lower than the remaining samples. In addition, they were characterized by higher *ε*’ and *σ* (measured at 4.0 MHz as a midway frequency value), which are inversely related to the porosity values (Figure 13).

### 3.8. Industrial Applications

The highly porous nature of the studied ceramic samples, as produced from both arc furnace slag and sludge waste mixtures, suits them for creation of building materials such as wall tiles. The relatively low electric conductivity, imaginary electric modulus, and dielectric constant values of the studied samples indicate their applications as poor to fair semiconductors (at 50 Hz and higher frequencies). The increasing iron oxide content with increasing arc furnace slag percentages (from 1.6% for AFS1 to 7.04% for AFS9) reduces the possibility of this application by increasing electric activity. In addition, the presence of certain impurities in these samples increases their ability to conduct electric charges on the order of μS (Khater et al., 2019, 2020, Dimitrijev, 2011) [35,36,56].

From an economic point of view, lightweight porous ceramic prepared from recycling hazardous waste materials tends to cost less than porcelain and is much lighter. It is often used for wall and ceiling installations. However, there are some significant restrictions with this material: it is not as strong as porcelain, so it does not make the best walking surface; it can be freezing underfoot in the winter; and heavy tile can be challenging to install. Therefore, this kind of ceramic is a good candidate for wall tile applications.

Porous ceramic wall tile bodies, whose microstructure and composition differ entirely from those of porcelain bodies (higher porosity and smaller glassy phase content), can be incredibly affordable and widely used worldwide. All these requirements are highly fulfilled in this work through the recycling of both electric arc furnace slag and ceramic sludge waste.

In this work, cleaning the environment from industrial waste byproducts was our first goal. The production of lightweight ceramic materials via a low energy consuming process to improve our environment and reduce the headache caused by global warming was our second target.

## 4. Conclusions

1. Cleaning the environment via waste management of nonrenewable products is very important for our daily life. In the present work, lightweight porous ceramic materials composed of wollastonite (β-wollastonite or parawollastonite), gehlenite and low quartz phases were successfully prepared through recycling two industrial wastes, i.e., arc furnace slag and ceramic sludge. The recycling process consumed little energy, and therefore can be easily applied on pilot and industrial scales.
2. The formed phases depended on the CaO/SiO_2_ ratio in both waste materials. Lower CaO/SiO_2_ content led to the formation of β-wollastonite and quartz phases with small amounts of the gehlenite phase. A higher CaO/SiO_2_ ratio led to the formation of parawollastonite and gehlenite phases but hindered the development of the low quartz phase.
3. By applying different sintering temperatures to a selected sample (AFS5), it was found that both polymorphic structures of wollastonite were formed, either β-wollastonite (unstable) or parawollastonite (stable). β-wollastonite was converted into parawollastonite at high temperatures. By increasing the sintering temperature to 1150 °C, traces of the fayalite phase (Fe_2_SiO_4_) developed.
4. The porosity of the prepared materials was affected by sintering temperatures. The higher the temperature, the lower the porosity, due to the formation of a compacted microstructure.
5. The porosity, density and electrical properties of prepared materials depended on the composition of starting materials and formed phases. Increases in CaO and Al_2_O_3_ was accompanied by increasing porosity, while increases in MgO and Fe_2_O_3_ led to decreasing porosity and increasing dielectric constant and electric conductivity.
6. The selected sample sintered at 1100 °C exhibited lower dielectric parameters than those sintered at 1050 °C and 1150 °C.

## Figures and Tables

**Table 1:** Chemical analysis of used raw materials.

Oxide	Arc Furnace Slag	Ceramic Sludge	Limestone	Silica Sand
(wt.%)				
SiO_2_	16.09	61.89	0.15	99.2
CaO	38.59	6.58	55.7	0.1
Al_2_O_3_	5.24	17.28	0.22	0.28
MgO	14.62	0.9	0.1	trace
Na_2_O	0.62	1.83	trace	trace
BaO	0	0.64	nil	trace
MnO	0.74	0.02	nil	trace
Fe_2_O_3_	10.29	0.99	0.03	0.03
TiO_2_	0.16	0.74	nil	trace
K_2_O	0.13	1.24	trace	trace
L.O.I.	11.15	5.86	44.02	0.4

**Table 2:** Chemical compositions and corresponding batch percentages of investigated samples.

Batch No.	Nominal Composition (wt.%)		Calculated Oxide Constituents (wt.%)					CaO/SiO_2_	Batch Ingredients (wt.%)			
	AF	S	SiO_2_	CaO	Al_2_O_3_	Fe_2_O_3_	MgO		Arc Furnace Slag	Sludge	Limestone	Silica Sand
AFS1	10	90	47.86	32.73	13.42	1.6	1.9	0.68	6.52	58.64	34.84	0
AFS2	20	80	47.8	30.22	13.48	2.58	3.3	0.63	14.6	58.39	27.08	0
AFS3	30	70	47.65	27.35	13.53	3.71	4.97	0.57	24.84	57.95	17.21	0
AFS4	40	60	47.52	23.79	13.61	5.14	6.97	0.5	38.32	57.48	4.2	0
AFS5	50	50	52.58	21.46	10.7	5.37	7.37	0.41	42.91	42.91	0	14.17
AFS6	60	40	48.54	24.52	9.56	6.25	8.69	0.51	51.37	34.25	0	14.38
AFS7	70	30	48.62	25.55	7.81	6.61	9.25	0.52	55.74	23.88	0	20.38
AFS8	80	20	49.44	26.02	6.19	6.82	9.6	0.53	58.71	14.68	0	26.61
AFS9	90	10	49.8	26.62	4.85	7.04	9.97	0.53	61.64	6.85	0	31.51

AF = arc furnace slag, S = ceramic sludge.

**Table 3:** Main resulting phases and values of porosity and density for the investigated ceramic samples after being treated at 1100 °C for 1 h.

Sample No.	Density g/cm^3^	Porosity%	Phases Developed
AFS 1	1.896	41.12	β-woll., Geh., QZ-low
AFS 2	1.906	40.14	Parawoll., Geh., QZ-low
AFS 3	1.875	38.87	Parawoll., Geh., QZ- low
AFS 4	1.824	41.66	Parawoll., Geh., QZ-low
AFS5	2.01	31.16	β-woll., Geh., QZ-low
AFS 6	2.211	29.4	Geh., β-woll., QZ-low
AFS 7	2.203	29	β-woll, Geh., QZ-low
AFS 8	2.106	28.41	β-woll., Geh., QZ-low
AFS 9	1.798	47.26	β-woll., Geh., QZ-low

Parawoll. = parawollastonite, β-woll. = β-wollastonite, Geh. = gehlenite, Qz-low = low quartz.

**Table 4:** Effect of sintering temperatures on density, porosity and phases developed for sample AFS5.

Sintering Temperatures	Density g/cm^3^	Porosity (%)	Phases Developed
1050 °C	1.691	43.98	Parawoll.,Geh., QZ-low
1100 °C	2.010	31.16	β-woll., Geh., QZ-low
1150 °C	1.990	33.36	Parawoll, Fay., QZ-low

Parawoll. = parawollastonite, β-woll. = β-wollastonite, Geh. = gehlenite, Fay. = Faylite, Qz-low = low quartz.

## Data Availability

The data of this work is available for first author after publication.
